# Comparative efficacy of BioMin-F, Colgate Sensitive Pro-relief and Sensodyne Rapid Action in relieving dentin hypersensitivity: a randomized controlled trial

**DOI:** 10.1186/s12903-021-01864-x

**Published:** 2021-10-06

**Authors:** Saba Arshad, Syed Jaffar Abbas Zaidi, Waqas Ahmed Farooqui

**Affiliations:** 1 Department of Oral Biology, Dr. Ishrat-Ul-Ebad Khan Institute of Oral Health Sciences (DIKIOHS), Dow University of Health Sciences, Karachi, Pakistan; 2 Department of Oral Biology, Dow Dental College, Dow University of Health Sciences, Karachi, Pakistan; 3 Department of Research, School of Public Health, Dow University of Health Sciences, Karachi, Pakistan

**Keywords:** Dentine hypersensitivity, Fluoro-calcium phospho-silicate, Arginine, Strontium acetate

## Abstract

**Background:**

Dentin hypersensitivity (DH) is a sharp toothache that influences a patients' oral health-related quality of life. Oral dentifrices have been marketed for pain relief within a minute for DH. The permanent management of DH is being investigated with the remineralisation potential of bioactive agents in dentinal tubules. This study investigated the relief from pain in DH in one minute after applying over the counter (OTC) dentifrices with Pro-Argin™ and strontium acetate and directly compared them with fluoro-calcium phospho-silicate (FCPS)-based dentifrices for immediate and sustained inhibition of painful stimulus provoking DH.

**Methods:**

A randomised, controlled, triple-blinded clinical trial was conducted with 140 participants clinically diagnosed with DH and equally randomized into four groups with parallel treatment assignment of FCPS, Pro-Argin™, 8% strontium acetate, and sodium fluoride-based OTC dentifrices, and tested for DH with air blast, mechanical, and water jet stimuli on SCHIFF cold air sensitivity scale (SCASS) and visual analogue scale (VAS) at interim efficacy intervals of one minute, three days, two, four, and six weeks, subsequently.

**Results:**

A total of 128 participants completed the trial. All the treatment groups showed statistically significant improvement in DH with *p* < 0.001 relative to baseline at all time points. Pro-Argin™ showed a greater reduction in DH with mean scores of (1.34 ± 0.68) (4.20 ± 1.70) (3.05 ± 2.17) followed by strontium acetate (1.57 ± 0.81) (4.65 ± 1.87) (3.75 ± 1.97) on SCASS and VAS for mechanical and water jet stimuli, one minute after application. There was no statistically significant treatment difference between the two (*p* = 0.499). FCPS showed the highest reduction in DH on SCASS and VAS for waterjet stimuli with mean scores of (0.97 ± 0.68) (1.80 ± 1.73) and Pro-Argin™ on VAS for mechanical stimuli with mean scores of (2.15 ± 1.92) in six weeks.

**Conclusion:**

OTC dentifrices with Pro-argin™ and strontium acetate are effective for immediate pain relief from DH, and FCPS could be the best possible treatment option for long term management of DH.

***Trial registration*:**

ID: NCT04249336 (https://clinicaltrials.gov/ct2/show/NCT04249336), Date of Registration: January 30, 2020 (Retrospectively registered).

## Background

Dentine hypersensitivity (DH) is a chronic condition with acute, transient pain in non-carious teeth that originates in response to thermal stimuli such as hot or cold, chemicals like acidic or sweet or salt, and mechanical stimuli due to exposure to dentinal tubules without any apparent clinical disease [1]. The main etiological factors are abrasion and erosion that may or may not be associated with gingival recession in DH. DH most commonly involves the facial surfaces of maxillary and mandibular permanent teeth. Canines and premolars are the most commonly affected teeth among 25–30% of the adult population [2].

The prevalence of DH was found to vary from about 5 to 62% owing to the use of different diagnostic and measuring tools and age groups in the published literature, with the best average estimate of 34% worldwide [3]. It remains a prevalent global disease with two different treatment modalities of home-care desensitisation with the over-the-counter (OTC) desensitisers such as potassium, fluorides, arginine, strontium, and bioactive glasses (BAG) [4] and in-office application of bioactive formulations such as glutaraldehyde, resin-based bonding agents and restorative materials, amorphous calcium phosphate based-tooth mousse, and lasers in the dental clinics [5]. Though all bioactive agents have a significant treatment effect in reducing DH, there is currently no consensus on the unequivocal efficacy of any product or bioactive agent used for managing the condition due to huge variations and heterogeneity in the conduct of clinical trials on DH [6].

Oral dentifrices have been marketed extensively for pain relief in one minute after topical application on sensitive teeth, with the claims of several dentists recommending the formulations containing Pro-argin™ and 8% strontium acetate. However, strontium acetate can potentially obliterate dentinal tubules by replacing the calcium ions of hydroxyapatite crystal lattice structure with strontium ions along with its nerve depolarisation treat DH [7]. In comparison, the Pro-argin™ can make mechanical barrier of calcium phosphate precipitates on exposed dentinal tubules up to 2 µm depth by the interaction of positively charged arginine, amino acids and type 1 collagen fibres [8].

A meta-analysis by Grünberg et al. [9] stated in 2017 that arginine and strontium acetate-based desensitisers had equal clinical significance in reducing pain in DH and the other findings of the meta-analysis suggested that strontium acetate and Pro-argin™ should be compared for clinical efficacy immediately after topical application by independent health authorities. Another meta-analysis by Hu et al. [10] examined the evidence for strontium acetate and Pro-argin™ in 2018, and their findings depicted low quality of evidence for strontium acetate on the Grading of Recommendations, Assessment, Development, and Evaluations system (GRADE).

The prime focus of treatment nowadays is based on the exploration of novel materials to remineralise the exposed tubular endings and mimic and restore the structure of the dentin [11]. Today's era of flourishing research in DH is based on incorporating bioactive ingredients in commercially available oral dentifrices that can induce deep intratubular remineralisation of dental hard tissues for permanent or long-term treatment purposes. A previously published study by Samueli et al. [12] suggested a novel BAG component of fluoro-calcium phospho-silicate (FCPS)-containing OTC dentifrice, with the potential of fluorapatite-like crystals formation in dentin in 2017. FCPS was incorporated in OTC dentifrice with calcium, low contents of fluoride, and high contents of phosphate ions. These ingredients resulted in rapid fluorapatite crystallisation at exposed tubular ends of dentin [12]. These fluorapatite crystals were less susceptible to acidic dissolution than hydroxyapatite crystals and thus were more desirable [13]. In vitro analysis suggested that FCPS-based dentifrice enabled the slow and sustained release of fluoride contents, resulting in more stable fluorapatite crystals in saliva [14, 15].

Pessoa, Loretto et al. found the clinical efficacy of Pro-argin™ better than strontium acetate for mechanical and air-blast stimulated DH on VAS and SCASS in follow-up periods of one minute, three days, two, four and eight weeks in a systematic review in 2015. However, they also suggested further comparative evaluations of two dentifrices with large sample sizes as limited scientific evidence was found in previous studies [16].

Researchers have investigated the clinical efficacy of popular propriety occluding dentifrices containing Pro-argin™, strontium acetate, and FCPS formulations for treating DH and have found no adverse effects. Therefore, this trial was planned to investigate the clinical efficacy of Pro-argin™ and strontium acetate for immediate pain relief in DH after one minute of topical application to fill the research gap associated with limited published evidence and risk of bias in selection, performance, attrition, and reporting as predicted by Pessoa et al. [16] in a systematic review in 2015.

Moreover, FCPS as a new bioactive material revealed promising potency of sustainable management of DH in a previously conducted trial by Ashwini et al. in 2018 [17]. A head-to-head comparative evaluation of these most effective bioactive agents, such as arginine and BAG-based desensitisers, was suggested by Martins et al. [18] in a meta-analysis in 2020. Hence, the effectiveness of clinically available formulations of FCPS in OTC dentifrices needs further exploration for the permanent management of DH.

Based on the outcomes of previous systematic reviews and meta-analysis, the rationale of the present study was to substantiate the claim of immediate pain relief after one-minute application in DH by non-invasive surface application of OTC dentifrices containing Pro-argin™in Colgate® Sensitive Pro-Relief™, and 8% strontium acetate in Sensodyne Rapid Action™ and other objective was the comparative evaluation of the clinical efficacy of three technologies, BAG-based FCPS in BioMin^R^ F, Pro-argin™, and 8% strontium acetate at efficacy intervals of day three, week two, four and six for sustained inhibition of painful stimulus provoking DH as all three are dentinal tubular occluding dentifrices. Therefore, the first null hypothesis was that mean scores for pain due to DH among different treatment groups for immediate relief remain. Therefore, it was considered to be rejected at a *p *value ≤ of 0.05. The second null hypothesis was that mean scores for pain due to DH among different treatment groups for sustained relief remains the same, and it was considered to be rejected at a *p *value ≤ 0.05.

## Methods

This trial was registered in an ICMJE approved registry at https://clinicaltrials.gov/) under protocol Identifier: NCT04249336 [19] following the CONSORT guidelines [20] for parallel-group randomized trials after approval from the Institutional Review Board of Dow University of Health Sciences with reference no. IRB-1351/DUHS/Approval/2019/184.

### Study design and settings

A randomised controlled, triple-blinded, phase-3 clinical trial was conducted at the Department of Periodontology at Dr Ishrat-ul-Ebad Khan Institute of Oral Health Sciences (DIKIOHS) in Dow University of Health Sciences (DUHS), Karachi, Pakistan from September 2019 to December 2020. Participants were randomly allocated to four treatment arms and randomly assigned with OTC dentifrices having FCPS, Pro-argin™, strontium acetate, and sodium fluoride (NaF) with an allocation ratio of 1:1:1:1.

### Sample size calculation

**Fig. 1:**
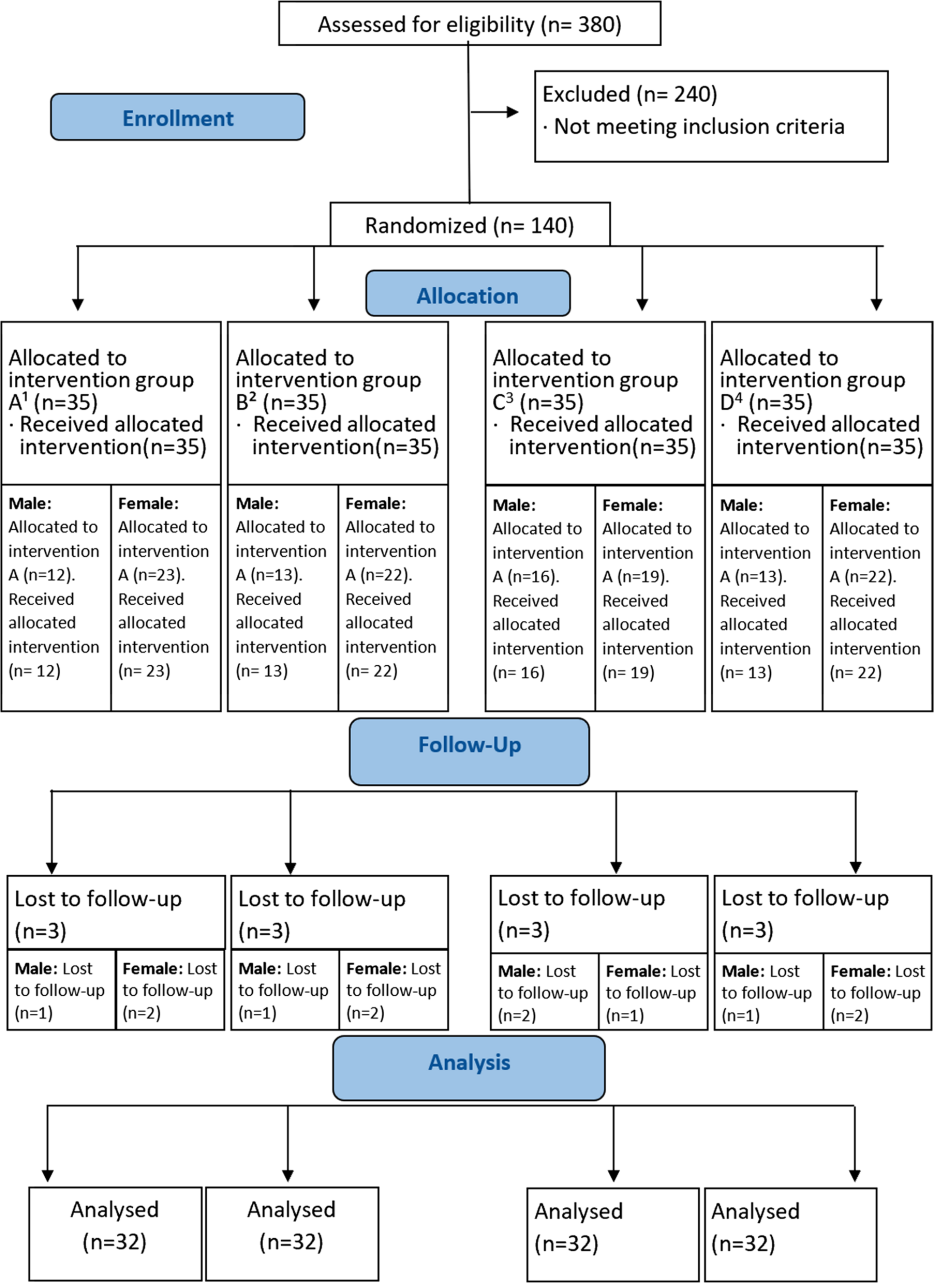
CONSORT flow chart. ^1^Intervention for Group A denotes BioMin F. ^2^Intervention for Group B denotes Colgate Sensitive Pro relief™. ^3^Intervention for Group C denotes Sensodyne Rapid Action™. ^4^Intervention D denotes Colgate Total™

The total sample size was 140 participants, with thirty-five in each group. We were using the PAS version.11, two independent sample t-test with 95% confidence interval and 80% power of test, mean ± SD of air blast stimulus test score of test group 1.86 ± 0.41 and control group 2.22 ± 0.41 two weeks after baseline test score, the calculated sample size was 22 [21] which was raised to 35 including five as drop out for each group (Fig. 1).

### Patient eligibility and recruitment

The participants with self-reported sensitivity in teeth were clinically diagnosed with DH. A total of 140 met the eligibility criteria with at least two sensitive teeth anterior to molars due to erosions or abrasions with or without an associated gingival recession. In addition, they were responding to air-blast stimulus on the Schiff cold air sensitivity scale (SCASS) with scores ≥ 2 [22]. Written informed consent was obtained with a signed agreement plan to visit for six weeks at interim efficacy intervals of day three, week two, week four, and week six. Individuals either with dental caries, heavily restored, or cracked teeth, or with orthodontic or prosthetic appliances, or with localised or generalised gingivitis or pulpitis and heavy calculus or using any desensitising toothpaste or mouth wash or any other desensitising products up to six weeks before the study were excluded from this trial.

These participants were randomized equally by the principal investigator into four entitled treatment arms using computer-generated random sequence numbers, and treatments were allocated randomly using sequentially numbered, opaque, sealed, and stapled envelopes (SNOSE). The sealed envelope contained details of unique subject numbers assigned individually in ascending order, group titles, and treatment codes and was placed in the box. Participants were asked to pick up the envelope from the box. Treatments provided to the patients were OTC dentifrices marketed with the claim of immediate pain relief in DH. These dentifrices were extracted in a plain tube by the dental assistant from commercial packing and labelled with treatment codes to keep the principal investigator and participants blinded at the time of treatment assignment. The statistician was also kept blinded. (Table 1) (Fig. 1).

**Table 1:** The participant's allocation and treatment assignments

Treatment arms	No. of participants(N = 140)	Group titles	Treatments	Active ingredients	Treatment codes
Experimental	35	Group A	BioMin F®	Fluoro-calcium-phospho-silicates (FCPS)	1
Active comparator 1	35	Group B	Colgate® Sensitive Pro-Relief™	Pro-Argin™ with 8.0% arginine and 1450 ppm fluorides as sodium mono-fluoro-phosphate in calcium carbonate base	2
Active comparator 2	35	Group C	Sensodyne Rapid Action™	8% strontium acetate, 1040 ppm fluorides as sodium fluoride	3
Placebo	35	Group D	Colgate® Total	Sodium fluoride, sodium mono-fluoro-phosphate, dicalcium phosphate with 1150 ppm fluorides	4

A toothbrushing demonstration model from Nissin Company specification [PE-ORT002] was used to explain to the study participants about applying OTC dentifrices for one minute with the participant's finger. Modified bass brushing method was demonstrated on the same model before the first application of dentifrices in the dental clinic. Soft tissues were isolated with cotton rolls, and adjacent teeth were isolated with cotton pellets. Baseline scores were recorded to measure the intensity of pain due to DH on SCASS with scores ≥ 2 [22] against air blast stimulus with a triple syringe of the dental unit and on a linear VAS of 10 cm length with scores ≥ 4 cm [23], against mechanical stimulus with a dental probe and water jet stimulus with a triple syringe. OTC dentifrices were applied and massaged gently with the applicator on at least two sensitive teeth per subject at two different sites, including cementoenamel junction and exposed dentinal surface, by the doctor in the office during the first visit. Dentifrices were removed carefully after one minute of undisturbed application by rinsing the air lightly with an air syringe, and the subject was asked to spit. The first post-treatment measure of SCASS and VAS scores was obtained immediately after removing dentifrices from teeth. Then, the repeated measure was obtained after five minutes for previous pain recovery, and dentifrices were provided to the patient for self-application. The maximum sampling time was 15 min.

All participants were instructed to dry the tooth surface with the cotton ball and apply a paste of about half-inch length on the dried surface for one minute, then brush the teeth twice daily after breakfast and before sleep as per directions demonstrated on the study model. Participants were recalled at interim efficacy intervals of three days, two weeks, four weeks, and six weeks for assessment of sustained relief. SCASS and VAS were introduced on each visit as primary and secondary outcome measures, respectively.

The study's primary objective was to investigate pain relief in one minute with Pro-argin™ and strontium acetate in DH measured on SCASS as a primary outcome measure and VAS as a secondary outcome measure. The secondary objective of the study was to compare the clinical efficacy of Pro-argin™, strontium acetate, and FCPS for immediate and sustained treatment response in DH that was measured on SCASS as a primary outcome measure and VAS as secondary outcome measure at efficacy intervals of one minute, day-three, week two, four and six.

Participants were strictly advised to refrain from acidic food and drink intake at least four hours before following up visit. Oral dentifrices were provided to the subject free of cost. In addition, participants were asked to record the overall sensitivity of their day-to-day experience on provided VAS sheets reporting pain on brushing, taking hot or cold beverages, and rinsing with tap water for the six weeks of the study.

### Statistical analysis

Statistical Package for the Social Sciences software v.21.0. was used for statistical analysis. The primary analysis population could be described as 32 participants per treatment group after lost to follow-ups of 12 out of 35 enrolled in the six-week trial who responded to the air blast stimulus for the primary outcome measure of treatment response one minute after topical application and after three days and two, four and six weeks subsequently, on SCASS with scores of ≥ 2. Paired sample T-test was used to compute mean scores to observe change relative to baseline at each time point. In addition, one-Way ANOVA with Post Hoc Tukey for pair-wise comparison was used to compare treatment groups computing percent change from baseline with formula (post-application mean scores-baseline mean scores/baseline mean scores).

The secondary analysis population could be described as 20 participants per treatment group out of 35 enrolled in the six-week trial who responded to mechanical and water jet stimuli on VAS with scores of ≥ 4 as secondary outcome measures. Wilcoxon Signed-Rank Test was used to compute mean scores relative to baseline at each time point. Friedman Test was used to observe post-application change in treatment responses within the group for 6 weeks. Kruskal–Wallis test was used to compare treatment groups. The mean scores with repeated measures were statistically analysed at each time point for study outcomes. *p *values of < 0.05 were considered as statistically significant.

## Results

A total of 140 participants were initially enrolled and randomised into the four treatment arms, with thirty-five in each group. Thus, 128 participants completed the trial and were statistically analyzed for primary outcome measure of SCASS scores after lost to follow up (Fig. 1).

Table 2 presents descriptive statistics of the primary analysis population as the primary statistical analysis was performed for 47 males and 81 females of age ranging from 18 to 50 years with a mean age of 34.1 ± 8.9. There was statistically no significant difference in the baseline characteristics of gender, age, baseline mean scores of clinical parameters of SCASS, and VAS used for mechanical and water jet stimuli between the four treatment groups (*p* > 0.05).

**Table 2:** Baseline descriptive statistics of four treatment groups

Baseline characteristics	Group A	Group B	Group C	Group D	*p *value
Age (mean ± SD)	33.2 ± 8.8	34.4 ± 8.4	35.6 ± 9.6	33.1 ± 9.1	0.620^¥^
Gender n (%)					0.824^α^
Male	11 (34.3)	11 (34.3)	14 (43.7)	11 (34.3)	
Female	21 (65.6)	21 (65.6)	18 (56.2)	21 (65.6)	
Baseline mean scores for SCASS	2.46 ± 0.50	2.46 ± 0.50	2.46 ± 0.50	2.46 ± 0.50	> 0.99^¥^
Baseline mean scores for VAS with mechanical stimulus	6.75 ± 0.91	6.70 ± 1.03	6.70 ± 1.17	6.70 ± 1.03	0.998^>^
Baseline mean scores for VAS with water jet stimulus	6.70 ± 0.80	6.70 ± 0.80	6.70 ± 0.80	6.70 ± 0.80	0.624^>^

Group A received BioMin F™. Group B received Colgate Sensitive Pro relief™. Group C received Sensodyne Rapid Action™. Group D received Colgate Total™

*SD* standard deviation, *SCASS* Schiff cold air sensitivity scale, *VAS* visual analogue scale

^¥^One way ANOVA; ^α^Chi-square test; ^>^Mann–Whitney test; *p* values were considered significant at 0.05

Table 3 demonstrates the percent change in the mean scores of DH on SCASS relative to baseline within the group for each treatment arm. Post-application changes on SCASS were observed after one minute, three days, two, four, and six weeks, subsequently. There was a significant (*p* < 0.001) reduction of 45.7% with Pro-argin™, 37.6% with 8% strontium acetate, 18.1% with FCPS, and 15.2% with NaF-based dentifrices relative to baseline on SCASS after one-minute application on sensitive teeth. In contrast, there was a significant (*p* < 0.001) reduction of 61.1% with FCPS, 60.1% with Pro-argin™, 53.4% with 8% strontium acetate, and 25% with NaF-based dentifrices relative to baseline after subsequent six weeks of application observing sustained relief from DH on SCASS.

**Table 3:** The primary outcome measure of Immediate and sustained treatment response relative to baseline in DH using Schiff cold air sensitivity scale

Post-application efficacy intervals	Group A	Group B	Group C	Group D	*p *value
Immediate					< 0.001^¥^
(Mean scores ± SD)	2.03 ± 0.70	1.34 ± 0.68	1.57 ± 0.81	2.17 ± 0.61	
(*p *value)^β^	< 0.001	< 0.001	< 0.001	0.001	
Percent change from baseline (%)	18.1	45.7	37.6	15.2	
Day 3					0.016^¥^
(Mean scores ± SD)	1.91 ± 0.73	1.81 ± 0.59	1.81 ± 0.53	2.19 ± 0.60	
(*p *value)^β^	< 0.001	< 0.001	< 0.001	0.003	
Percent change from baseline (%)	22.9	25.5	24.4	10.2	
Week 2					< 0.001^¥^
(Mean scores ± SD)	1.25 ± 0.71	1.30 ± 0.58	1.56 ± 0.70	2.12 ± 0.68	
(*p *value)^β^	< 0.001	< 0.001	< 0.001	0.001	
Percent change from baseline (%)	48.9	46.9	37.2	14.2	
Week 4					< 0.001^¥^
(Mean scores ± SD)	1.03 ± 0.70	1.09 ± 0.62	1.33 ± 0.64	1.91 ± 0.71	
(*p *value)^β^	< 0.001	< 0.001	< 0.001	< 0.001	
Percent change from baseline (%)	58.5	55.8	45.4	22.5	
Week 6					< 0.001^¥^
(Mean scores ± SD)	0.97 ± 0.68	0.97 ± 0.68	1.18 ± 0.57	1.82 ± 0.67	
(*p *value)^β^	< 0.001	< 0.001	< 0.001	< 0.001	
Percent change from baseline (%)	61.1	60.1	53.4	25.0	

Group A received BioMin F™. Group B received Colgate Sensitive Pro relief™. Group C received Sensodyne Rapid Action™. Group D received Colgate Total™

*SD* standard deviation

^β^Paired sample T-test; ^¥^one Way ANOVA; *p *values were considered significant at 0.05

Table 4 demonstrates a comparison between treatment groups observing better clinical efficacy in managing DH on SCASS. Post-hoc analysis revealed a significant reduction in DH scores in treatment arms compared to control on completing the six-week trial (*p* < 0.05). However, there was no significant treatment difference between dentifrices containing Pro-argin™ and strontium acetate (*p* > 0.05) at each efficacy interval. However, Pro-argin™ formulation showed 8% more improvement in clinical symptoms of DH than strontium acetate formulation one minute after application. Moreover, there was no significant difference between dentifrices containing FCPS, Pro-argin™, and 8% strontium acetate (*p* > 0.05) on completion of trial after six weeks. However, FCPS formulation showed 1% more improvement than Pro-argin™ and 7% more than strontium acetate-based dentifrices.

**Table 4:** Comparison of treatment response between treatment groups on Schiff cold air sensitivity scale

Post-application efficacy intervals	Percentage difference between treatment groups (%age)					
	Groups A vs B	Groups A vs C	Groups A vs D	Groups B vs C	Groups B vs D	Groups C vs D
Immediate	− 27	− 19	2	8	30	22
(*p *value)^¥^	< 0.001	0.005	0.960	0.499	< 0.001	0.001
Day-3	− 1	− 2	12	1	15	14
(*p *value)^¥^	0.991	0.960	0.084	0.997	0.024	0.041
Week-2	1	11	34	9	32	23
(*p *value)^¥^	0.984	0.152	< 0.001	0.290	< 0.001	< 0.001
Week-4	2	13	36	1	33	22
(*p *value)^¥^	0.971	0.145	< 0.001	0.333	< 0.001	< 0.002
Week-6	1	7	36	6	35	28
(*p* value)^¥^	0.998	0.573	< 0.001	0.680	< 0.001	< 0.001

Group A received BioMin® F. Group B received Colgate Sensitive Pro-relief™

Group C received Sensodyne Rapid Action™. Group D received Colgate Total™

^¥^One way ANOVA: Post Hoc Tukey; *p* values were considered significant at 0.05

− value means the denominator group showed more clinical reduction in pain due to DH

+ value means numerator group showed more clinical reduction in pain due to DH

Table 5 presents the change in the mean scores of VAS against mechanical stimulus from baseline to subsequent efficacy interval within each treatment arm. Dentifrices-containing Pro-argin™ showed a significant reduction in DH with mean scores of (4.20 ± 1.70) followed by strontium acetate (4.65 ± 1.87) followed by FCPS (5.10 ± 1.88) one minute after application. After six weeks, Pro-argin™-based dentifrices showed greater clinical efficacy with mean scores (2.15 ± 1.92) than other treatment arms for mechanical stimulated DH.

**Table 5:** The secondary outcome measure of Immediate and sustained treatment response in DH on visual analogue scale using mechanical stimulus

Post application efficacy intervals	Group A	Group B	Group C	Group D	*p* value
Immediate					0.002^**π**^
(Mean scores ± SD)	5.10 ± 1.88	4.20 ± 1.70	4.65 ± 1.87	5.65 ± 1.49	
(*p *value)^ψ^	< 0.001	< 0.001	< 0.001	< 0.001	
Day 3					0.110^**π**^
(Mean scores ± SD)	4.90 ± 1.48	3.15 ± 1.95	3.90 ± 2.31	5.55 ± 1.46	
(*p *value)^ψ^	< 0.001	< 0.001	< 0.001	< 0.001	
Week 2					0.002^**π**^
(Mean scores ± SD)	3.45 ± 1.84	3.10 ± 2.19	3.60 ± 2.25	5.45 ± 1.57	
(*p *value)^ψ^	< 0.001	< 0.001	< 0.001	< 0.001	
Week 4					0.001^**π**^
(Mean scores ± SD)	2.90 ± 2.07	2.45 ± 2.16	3.10 ± 2.19	5.00 ± 1.68	
(*p *value)^ψ^	< 0.001	< 0.001	< 0.001	< 0.001	
Week 6					0.001^**π**^
(Mean scores ± SD)	2.35 ± 2.00	2.15 ± 1.92	3.05 ± 2.04	4.55 ± 1.95	
(*p *value)^ψ^	< 0.001	< 0.001	< 0.001	< 0.001	
(*p *value)^ω^	< 0.001	< 0.001	< 0.001	< 0.001	

Group A received BioMin® F. Group B received Colgate Sensitive Pro relief™. Group C received Sensodyne Rapid Action™. Group D received Colgate Total™

*SD* standard deviation

^**ψ**^Wilcoxon Signed-Rank Test; ^**π**^Kruskal–Wallis test; ^**ω**^Friedman test; *p* values were considered significant at 0.05

Table 6 presents the change in the mean scores on VAS against waterjet stimulus from baseline to subsequent efficacy interval within each treatment arm. Dentifrices-containing Pro-argin™ showed a significant reduction in DH with mean scores of (3.05 ± 2.17) followed by strontium acetate (3.75 ± 1.97) followed by FCPS (5.30 ± 1.83) one minute after application. After six weeks, FCPS-based dentifrices showed greater clinical efficacy with mean scores (1.80 ± 1.73) than other treatment arms for waterjet stimulated DH.

**Table 6:** The secondary outcome measure of Immediate and sustained treatment response in DH on visual analogue scale using water jet stimulus

Post application efficacy intervals	Group A	Group B	Group C	Group D	*p* value
Immediate					< 0.001^**π**^
(Mean scores ± SD)	5.30 ± 1.83	3.05 ± 2.17	3.75 ± 1.97	5.33 ± 1.93	
(*p *value)^ψ^	< 0.001	< 0.001	< 0.001	< 0.001	
Day 3					0.045^**π**^
(Mean scores ± SD)	4.75 ± 2.26	4.05 ± 1.96	4.60 ± 1.56	5.33 ± 1.90	
(*p *value)^ψ^	< 0.001	< 0.001	< 0.001	< 0.001	
Week 2					< 0.001^**π**^
(Mean scores ± SD)	3.50 ± 1.90	3.14 ± 2.07	3.60 ± 1.46	5.10 ± 1.94	
(*p *value)^ψ^	< 0.001	< 0.001	< 0.001	< 0.001	
Week 4					< 0.001^**π**^
(Mean scores ± SD)	2.30 ± 1.94	2.73 ± 21.98	3.05 ± 1.63	4.67 ± 2.03	
(*p *value)^ψ^	< 0.001	< 0.001	< 0.001	< 0.001	
Week 6					< 0.001^**π**^
(Mean scores ± SD)	1.80 ± 1.73	2.27 ± 1.98	2.75 ± 1.51	4.33 ± 2.03	
(*p *value)^ψ^	< 0.001	< 0.001	< 0.001	< 0.001	
*P *value	< 0.001^**ω**^	< 0.001^**ω**^	< 0.001^**ω**^	< 0.001^**ω**^	

Group A received BioMin® F. Group B received Colgate Sensitive Pro relief™. Group C received Sensodyne Rapid Action™. Group D received Colgate Total™

*SD* standard deviation

^**ψ**^Wilcoxon Signed-Rank test; ^**π**^Kruskal–Wallis test; ^**ω**^Friedman test; *p* values were considered significant at 0.05

No adverse events like gingival inflammation, bad taste, allergies, fluoride incompatibility, and dental or tongue stains were observed for all dentifrices.

## Discussion

All the treatment groups subjected to testing in the present study revealed a considerable clinically significant symptomatic reduction in DH relative to the pre-treatment condition that was also statistically significant. In addition, the frequency of participants diagnosed with DH using air blast stimulus was observed as 36.8% at the time of enrolment in the six-week clinical trial that was found consistent with findings of a survey report demonstrating 36.4% frequency of DH among adults in Karachi, Pakistan [24].

The study's primary objective was to investigate pain relief in one minute after topical application of Pro-argin™ and strontium acetate on sensitive teeth in DH. It was measured by assaying change in SCASS scores of pains with Pro-argin™ and 8% strontium acetate after one minute of topical application on the sensitive teeth. Both treatments showed clinically and statistically significant (*p* < 0.001) relief in pain due to DH relative to baseline on immediate post-treatment observation, as shown in Table 3. In our study, Pro-argin™ treatment revealed an immediate clinical reduction of 45.7% on SCASS, 56% on VAS used for mechanical stimuli, and 48.1% on VAS used for water jet stimuli in DH demonstrated in Tables 3, 5, and 6. These findings were similar to a previous study conducted by Schiff et al. [22] reporting the instant relief from DH by 44.1% with Pro-argin™ on SCASS. Another study by Vu Pham and Anh Nguyen reported clinical improvement in DH by 38.9% on SCASS and 40.2% on VAS for mechanical stimulated DH immediately after application of Pro-argin™ [25]. Results of our study, together with the results of the previous studies, confirm the immediate clinical efficacy of Pro-argin™ for the relief of DH. Moreover, the occluding potential of Pro-argin™ has also been ascertained in a previous in vitro analysis conducted by Lavender et al. [8] that demonstrated the inherent ability of chemical interaction between arginine and type 1 collagen molecules in the salivary alkaline PH, thus resulting in endogenous calcium phosphate deposition on exposed dentinal tubules.

The next study outcome of our study was related to 8% strontium acetate with clinical reduction of 37.6% on SCASS and 46% on VAS from baseline using mechanical and water jet stimuli immediately after topical application on sensitive teeth as depicted in Tables 3, 5, and 6. The outcomes are in concordance with the previous studies conducted by Layer and Hughes [26], Zang and Shaw [27], and Mason et al. [28] reporting the immediate effect of 8% strontium acetate in alleviating clinical symptoms of DH with clinically and statistically significant (< 0.001) measures relative to baseline [26]. Hence, the current study outcomes, along with previously published findings, corroborate the in vitro findings of obliteration of dentinal tubules with strontium acetate impeding painful fluid movement in dentine [29].

The comparative evaluation was done for the clinical efficacy of Pro-argin™ and 8% strontium acetate, and a statistically significant (*p* > 0.05) difference was not found between these two formulations in the current study. However, Pro-argin™ showed more clinical improvement in air blast and mechanically stimulated DH compared to 8% strontium acetate, as shown in Tables 4 and 5. While 8% strontium acetate showed a greater clinical reduction in water jet stimulated DH on VAS than Pro-argin™ at all efficacy intervals, as demonstrated in Table 6. Besides, these findings of the current study could be related to previously conducted clinical trials indicating the better clinical effectiveness of Pro-argin™ than strontium acetate against air-blast and tactile stimulus after three days and one week of application [30, 31]. A meta-analysis by Yang et al. [32] reported statistically significant improvement in mechanical stimulated DH with Pro-argin™ immediately after topical application relative to 8% strontium Acetate in 2016. Hu et al. [33] again reported a 54% clinical reduction in DH with Pro-argin™ in eight weeks clinical trial using SCASS. A study evaluating the efficacy of strontium acetate relative to arginine-based dentifrices was conducted by West et al. It revealed an immediate improvement with strontium acetate more than Pro-argin™ on SCASS. However, the manufacturers of the tested dentifrices were acknowledged in the study [34], so information bias was expected in the conducted trial. However, we can conclude that both Pro-argin™ and strontium acetate-based dentifrices disrupt the pathophysiology of DH and are effective for immediate pain relief even one minute after application on sensitive teeth.

We observed the varying treatment response at different efficacy intervals that could be due to the varying degree of patient's response to pain-provoking stimuli in DH. Therefore, in the current study, we used a dental explorer and the air blast and water jet stimuli from the triple syringe of the dental unit for testing DH to control the subjective variations in treatment response. According to Holland's guidelines of conducting a trial for managing DH, two diagnostic tools are sufficient for quantitative assessment of the clinical efficacy of desensitisers. In addition, they should be reproducible and clinically relevant to stimuli triggering pain in DH [35].

The second objective of the current study was to directly compare the clinical efficacy of FCPS, Pro-argin™ technology, and strontium acetate-based dentifrices due to their similar mode of action and tubular occluding potential in managing DH [36, 37].

Pro-argin™ resulted in the immediate treatment response and statistically significant (*p* < 0.001) clinical reduction in DH than FCPS in the present study, as shown in Table 3. The findings agreed with another in vitro trial by Mahmoodi et al. who examined FCPS and Pro-argin™ formulations in 2018. They explored greater tubular occlusion potential and more acid resistance of Pro-argin™ in comparison to FCPS after subsequent seven days of application on dentinal discs [38].

The current study outcomes showed significant decrease in pain scores of air-blasts and water jet stimulated DH with FCPS based dentifrices compared to Pro-argin™ and 8% strontium acetate after two, four, and six weeks subsequently, as depicted in Tables 4 and 5. These outcomes were found in concordance with the previous controlled clinical trial conducted by Patel et al. in 2019 using BioMin® F and Pro-argin™. They reported more clinical efficacy with BioMin™ than Pro-argin™ reduction in air-blast stimulated DH on VAS after one month [39].

We found more change in pain scores of VAS used for mechanical stimuli with Pro-argin™ than FCPS as shown in Table 5 that could be relatable with a randomised controlled trial of FCPS and arginine based desensitizers reporting better treatment response of arginine than FCPS in the cold stimulated DH after two weeks to one month, subsequently [40].

The probable reason for variations between this clinical trial and previously conducted studies could be attributed to racial or ethnic differences between the conducted trials. Our study included an Asian population, and most of the published literature was associated with the Caucasian population. In our study, patient compliance related to the recommended time of application and method of the application provided by doctors was controlled by subjecting multiple efficacy intervals and different methods for assessment of the change in pain scores due to DH. A meta-analysis by Matranga et al. [41] was performed in 2017 and reported inappropriate statistical methodologies related to the normal distribution of data and equality of variances, along with lacking information in 77.1% of the thirty-five randomised trials on DH management. Therefore, the current study outcomes were analysed by parametric and non-parametric statistical tests after assessing the normal distribution of data and equality of variances with Shapiro Wilk statistics.

The strength of this study was that participants were randomised into four treatment groups to generalise the results for the population [32]. Participants analyzed per group were more than thirty fulfilling the requirement of Holland's guidelines for conducting clinical trials for the management of DH [42]. Published literature [16, 31, 43] has determined that all previous trials on treating DH were conducted on smaller samples, which might have prevented the results from being extrapolated on the general population.

The clinical significance of this study can be highlighted by the fact that this was a person-centred clinical trial where patients were the prime focus where the patients suffering from DH. We provided oral hygiene instructions and ways of relieving their pain from DH to improve their quality of life. In addition, patients were educated regarding the cause of the disease for prevention and awareness of oral hygiene measures, brushing techniques, and dietary influences in DH. These measures facilitated behaviour modification together with the promotion of knowledge and understanding in the society about DH.

The findings have revealed that both statistically and clinically significant change in mean scores of DH was observed with Pro-argin™ and 8% strontium acetate with *p* < 0.05 relative to baseline for immediate and sustained relief from DH using clinical parameters of SCASS and VAS. The statistically significant change in mean scores of DH was observed with FCPS with *p* < 0.05 relative to baseline for sustained relief from DH at follow-ups on day 3, week 2, week 4, and week 6, using clinical parameters of SCASS and VAS. The treatment difference between Pro-argin™ and 8% strontium acetate was not statistically significant with a *p* > 0.05 for immediate and sustained pain relief in DH and statistically significant relative to control on all clinical parameters of the SCASS and VAS. Treatment difference between FCPS, Pro-argin™, and 8% strontium acetate was not statistically significant with a *p* > 0.05 for sustained pain relief in DH. It was found statistically significant relative to control on all clinical parameters of the SCASS and VAS.

The limitation of our study is that fluoridated dentifrice was used as a control which, although regarded as not possessing any desensitising effects. However, there is a substantial possibility that these fluoridated dentifrices can reduce DH. This clinical trial has not included a run-in or washout period before the test phase due to time constraints and compliance of the potential participants. The dropouts of the participants at each efficacy interval may be considered the weakness of this trial that was due to COVID-19 lockdown. The impact of FCPS, Pro-argin™, and strontium acetate-based treatments of DH on the oral health-related quality of life together with the difference in the effectiveness of treatments were assessed individually rather than in groups in the current six-week trial. In this way, variability in individuals' pain response was tried to be controlled and will be discussed in part 2 of this manuscript.

More robust trials with a large number of participants are recommended for further assessing the impact of DH on the oral health-related quality of life and the difference in the effectiveness of treatments**.**

## Conclusion

Based on the synthesis of study results, we can conclude that Pro-argin™ in Colgate Sensitive Pro-relief™ and 8% strontium acetate in Sensodyne Rapid Action™ both are effective for pain relief in one minute after application in DH with better immediate treatment response of Pro-argin™ than strontium acetate. While FCPS in BioMin® F can be the best possible treatment option for long-term management of DH in the form of OTC dentifrices.

## Data Availability

The datasets analysed during the current study are available from the corresponding author on reasonable request.

## Abbreviations

DH: Dentin hypersensitivity
BAG: Bioactive glass
OTC: Over the counter
SCASS: Schiff cold air sensitivity scale
VAS: Visual analogue scale
NaF: Sodium fluoride
SPSS: Statistical Package for social sciences
ITT: Intention to treat
GRADE: Grading of Recommendations, Assessment, Development and Evaluations
